# Variation in type and frequency of diagnostic imaging during trauma care across multiple time points by patient insurance type

**DOI:** 10.1186/s12880-016-0146-8

**Published:** 2016-11-03

**Authors:** Nathaniel Bell, Laura Repáraz, William R. Fry, R. Stephen Smith, Alejandro Luis

**Affiliations:** 1 College of Nursing, University of South Carolina, 1601 Greene Street, Columbia, SC 29208 USA; 2 Department of Surgery, Good Samaritan Medical Center, Lafayette, CO USA; 3 Professor of Surgery, Trauma Medical Director, University of Florida, Gainesville, FL USA; 4 Palmetto Health Surgical Specialists, Columbia, SC USA

**Keywords:** Diagnostic imaging, Health equity, Wounds and injuries, Trauma centers

## Abstract

**Background:**

Research has shown that uninsured patients receive fewer radiographic studies during trauma care, but less is known as to whether differences in care are present among other insurance groups or across different time points during hospitalization. Our objective was to examine the number of radiographic studies administered to a cohort of trauma patients over the entire hospital stay as well as during the first 24-hours of care.

**Methods:**

Patient data were obtained from an American College of Surgeons (ACS) verified Level I Trauma Center between January 1, 2011 and December 31, 2012. We used negative binomial regression to construct relative risk (RR) ratios for type and frequency of radiographic imaging received among persons with Medicare, Medicaid, no insurance, or government insurance plans in reference to those with commercial indemnity plans. The analysis was adjusted for patient age, sex, race/ethnicity, injury severity score, injury mechanism, comorbidities, complications, hospital length of stay, and Intensive Care Unit (ICU) admission.

**Results:**

A total of 3621 records from surviving patients age > =18 years were assessed. After adjustment for potential confounders, the expected number of radiographic studies decreased by 15 % among Medicare recipients (RR 0.85, 95 % CI 0.78–0.93), 11 % among Medicaid recipients (0.89, 0.81–0.99), 10 % among the uninsured (0.90, 0.85–0.96) and 19 % among government insurance groups (0.81, 0.72–0.90), compared with the reference group. This disparity was observed during the first 24-hours of care among patients with Medicare (0.78, 0.71–0.86) and government insurance plans (0.83, 0.74–0.94). Overall, there were no differences in the number of radiographic studies among the uninsured or among Medicaid patients during the first 24-hours of care compared with the reference group, but differences were observed among the uninsured in a sub-analysis of severely injured patients (ISS > 15).

**Conclusions:**

Both uninsured and insured patients treated at a not-for-profit verified Level I Trauma Center receive fewer radiographic studies than patients with commercial indemnity plans, even after adjusting for clinical and demographic confounders. There is less disparity in care during the first 24-hours, which suggests that patient pathology is the determining factor for radiographic evaluation during the acute care phase. Results from this study offer initial evidence of disparity in diagnostic imaging across multiple insurance groups over different periods of trauma care.

## Background

The United States Census Bureau reported 42 million Americans, approximately 13.4 % of the population, were without health insurance in 2013 [1]. The Congressional Budget Office predicts that expanded health insurance coverage under the Affordable Care Act (ACA) will increase the number of nonelderly people with health insurance coverage to 33 million by 2016, reducing the number of uninsured by nearly 50 % [2]. However, the impact of the ACA will vary considerably as not all states will elect to expand Medicaid to close the insurance gap [3]. One perspective is that the ACA will have the paradoxical effect of increasing health disparities across states, particularly among racial minorities [4]. Initial evaluations of the health insurance uptake since the ACA substantiate this claim [5].

A growing body of literature has linked patient race/ethnicity and lack of insurance to an elevated risk of mortality following injury [6–12]. Commonly cited mechanisms for these disparities include poorer baseline health status [13], delayed or diminished access to emergent care after injury [14–18], more frequent inter-hospital transfers [19], as well as poorer access to comprehensive rehabilitation following care [20, 21]. Some studies suggest that variation in outcomes may be partially explained by injury cause, as minorities and uninsured patients disproportionately experience more lethal trauma [22]. However, that similar outcomes are found across medicine would suggest poorer outcomes are primarily mediated by socio-structural determinants as opposed to individual factors [23–26].

Some studies have examined whether variations in trauma outcomes can be attributed to the type and frequency of interventions patients receive during care. For example, Haas and Goldman (1994) found that uninsured trauma patients received fewer operative procedures and post-acute rehabilitative therapies than patients with private insurance or Medicaid [27]. Similarly, White et al. (2007) found that insured trauma patients received 68 % more radiological tests than those without insurance [28]. More recently, Bolorunduro et al. (2013) corroborated these findings with respect to variations in type and frequency of diagnostic imaging for pelvic fractures [29]. However, empirical data such as these are limited. In fact, there are few published studies as to whether differences in care exist across all insurance types rather than being specifically attributed to the uninsured. Furthermore, study findings have not always accounted for patient pathology. The purpose of this study is to examine a non-profit Level-I trauma hospital database to determine the independent effect of insurance status on the type and frequency of diagnostic imaging studies patients received at multiple points during care.

## Methods

This retrospective database analysis used data from the Palmetto Health Richland Hospital (PHRH) trauma registry. PHRH is an ACS verified Level-I tertiary care facility located in Columbia, South Carolina. PHRH trauma service attends to more than 2000 multiple-injured patients each year. The study was approved by the PHRH Institutional Review Board (IRB # Pro00034666) in accordance with the US Department of Health and Human Services (HHS) Protection of Human Subjects Regulations.

### Study population

Records from adult trauma patients (ages ≥ 18 years) between January 1, 2011 and December 31, 2012 that had been injured via blunt or penetrating trauma were included for analysis. Inclusion criteria for the PHRH trauma registry are a hospitalized stay ≥ 23 hours and primary diagnosis code of 800.9–959.9. Diagnostic codes for the trauma registry are defined using the *International Classification of Diseases, Ninth Revision, Clinical Modification* (ICD-9-CM) [30].

**Fig. 1:**
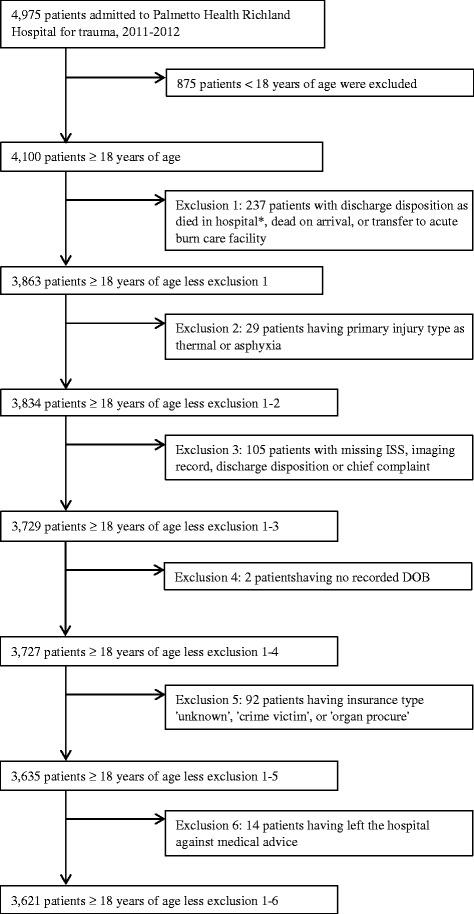
Study inclusion and exclusion criteria (*separately evaluated in a sub analysis)

Patients excluded from the registry include those with primary diagnosis codes of 905–909.9 (late effects of injury), 910.0–924.9 (superficial injuries, including blisters, contusions, abrasions and insect bites), 930.0–939.9 (foreign bodies), 885.5 and 888.9 (over the age of 65 with a same level fall), and 808.0–808.9, 820.0–820.9 (isolated hip fracture). Patients that died in hospital or during transit were excluded from the primary analysis. In-hospital deaths were included in a sub-analysis. Also excluded were those who were transferred to an acute burn facility or whose primary injury type was coded as thermal or asphyxia. Lastly, we excluded from the analysis all patient records missing a discharge disposition, injury severity score, no recorded age, or who left the hospital against medical advice (see Figure 1).

### Variables and coding

Our analysis included a subset of patient and clinical characteristics that have been previously reported to be important confounding factors for receipt of diagnostic imaging during trauma care [6, 27–29]. Demographic variables included patient sex, age, as well as race/ethnicity, coded as Caucasian (White), African American (Black), Hispanic, and Other by the registry. Patient insurance type was classified as: commercial indemnity insurance (auto, Blue Cross/Blue Shield, commercial, worker’s compensation, or managed care organization); Medicare; Medicaid; no insurance (medically indigent assistance program [MIAP] charity or self-pay); and Government (military/TRICARE, prison). All persons having an insurance status that did not fall into one of the defined categories, or that represented less than 2 % of the data set were excluded (crime victim [1.6 %], unknown [<1 %], organ procure [<1 %]). Clinical variables included Injury Severity Score (ISS), comorbidity, complications, injury mechanism, hospital length of stay (LOS), and Intensive Care United (ICU) admission.

Radiographic imaging was measured by comparing the number of studies ordered for each patient over their entire hospital stay and the number of studies ordered for each patient during the first 24-hours of care. Radiographic tests included all computed tomographic (CT) scans, all radiographic imaging, ultrasound, and x-ray imaging. A sub-analysis included the evaluation of the number and timing of the following CT scans: abdomen and pelvis with contrast, chest/abdomen/pelvis with contrast, cervical spine with reconstruction, facial bones without contrast, and head without contrast.

### Statistical analysis

In the unadjusted analysis, differences between means of continuous and normally distributed variables were compared using ANOVA. Differences between means of continuous variables that were not normally distributed were evaluated using the Kruskal-Wallis test. Differences in proportions of categorical variables were examined using *x* ^2^. Differences in median values were examined using the Hodges-Lehman estimate. Unadjusted and adjusted relative risk ratios (RR) were derived using negative binomial regression with a Pearson chi-square parameter to account for extra-Poisson variation. Sub-analyses were completed to determine if the relationship between insurance status and radiographic imaging persisted for those patients who died in hospital and when measured against more homogenous classifications of injury type and severity. All statistical analysis were performed using SPSS version 22 [31].

## Results

During the two year study period, the PHRH trauma registry captured 4975 adult patients hospitalized due to injury. A total of 3621 (66 %) records met the study inclusion criteria. In total, the primary payer source for 30.3 % of the patient population was commercial indemnity. Of the remaining patients, 20.4 % were covered by Medicare, 7.3 % by Medicaid, 35.6 % were uninsured, and 6.4 % were covered by Government insurance. The mean age was 45.8 years and 70.0 % of the patients were male (see Table 1). When grouped by race/ethnicity, 53.7 % of the patients were Caucasian, 39.9 % were African American, 3.6 % Hispanic, and 2.8 % defined as other.

**Table 1:** Demographic, clinical, and imaging characteristics of the patient population by insurance status

	Insurance status						
Characteristic	Overall	Commercial	Medicare	Medicaid	No Insurance	Government	*p* value
Diagnostic imaging, mean (SD)							
All imaging during hospital stay	15.1 (18.8)	17.3 (20.5)	13.5 (17.1)	20.6 (26.1)	13.8 (16.8)	10.8 (12.0)	<0.001
All imaging during first 24-hours	7.6 (6.7)	8.7 (7.5)	6.1 (5.7)	8.4 (7.4)	7.5 (6.3)	6.5 (5.7)	<0.001
CT scans within first 24-hours**	2.1 (1.9)	2.4 (1.9)	1.6 (1.7)	2.2 (2.0)	2.2 (2.0)	1.9 (1.8)	<0.001
Male, no. (%)	2528 (70.0)	743 (67.7)	374 (50.5)	163 (61.5)	1061 (82.4)	187 (81.0)	<0.001
Race/Ethnicity, no. (%)							
White	1944 (53.7)	698 (63.6)	532 (71.9)	99 (37.4)	482 (37.4)	133 (57.6)	<0.001
Black	1445 (39.9)	345 (31.4)	189 (25.5)	149 (56.2)	677 (52.6)	85 (36.8)	
Hispanic	132 (3.6)	25 (2.3)	5 (0.7)	7 (2.6)	88 (6.8)	7 (3.0)	
Other	100 (2.8)	29 (2.6)	14 (1.9)	10 (3.8)	41 (3.2)	6 (2.6)	
Age, mean (SD)	45.8 (19.8)	42.0 (16.5)	71.9 (14.1)	39.4 (15.6)	36.6 (12.1)	38.8 (16.3)	<0.001
ISS, mean (SD)	9.2 (7.5)	9.9 (7.6)	8.6 (6.1)	10.3 (9.4)	9.0 (7.6)	8.2 (7.0)	<0.001
Comorbidities (SD)	0.5 (0.8)	0.4 (0.7)	0.8 (0.9)	0.5 (0.8)	0.4 (0.7)	0.4 (0.7)	<0.001
Complications (SD)	0.71 (0.93)	0.70 (0.91)	0.77 (0.93)	0.90 (1.35)	0.67 (0.86)	0.55 (0.68)	<0.001
Blunt Injury, no. (%)	3058 (84.0)	975 (88.9)	700 (94.6)	217 (81.9)	977 (75.9)	189 (81.8)	<0.001
ICU admission (%)	1280 (35.4)	412 (37.6)	278 (37.6)	107 (40.5)	417 (32.4)	66 (28.6)	0.002
LOS* (IQR)	4 (7)	5 (9)	5 (7)	5 (14)	3 (7)	3 (5)	<0.001

*Median and interquartile range (IQR)

**CT scans of the abdomen and pelvis with contrast, chest/abdomen/pelvis with contrast, cervical spine with reconstruction, facial bones without contrast, and head without contrast

On average, patients received 15.1 radiographic images during their entire hospital stay. The mean number of images received during the first 24-hours was 7.6. The mean number of the selected CT scans received during the first 24-hours was 2.1. Additional clinical characteristics across insurance types are shown in Table 1. All demographic and clinical characteristics were statistically significantly different across insurance groups.

Table 2 shows the crude RRs for the total number of radiographic images received and the number of images received during the first 24-hours of care. In the total imaging model, all demographic and clinical characteristics except patient age and comorbidities were associated with the number of radiographic images patients received. Other exceptions were that the number of images varied among African American patients compared to Caucasian patients, but not among Hispanic and racial/ethnic groups classified as Other. All clinical and demographic variables were similarly associated with receipt of imaging within the first 24-hours with the exception of patient sex. Exceptions were found within the racial category, where differences were observed only among African American patients and among specific insurance types.

**Table 2:** Univariate analysis of the number of diagnostic images ordered and number of images ordered in the first 24 h

	Crude RR - All Imaging		Crude RR - Imaging < = 24-h	
Characteristic	RR (95 % CI)	*p*-value	RR (95 % CI)	*p*-value
Age	1.00 (1.00–1.00)	0.792	1.00 (1.00–1.00)	<0.001
Sex				
Female	[reference]	--	[reference]	--
Male	1.09 (1.00–1.19)	0.061	0.99 (0.92–1.05)	0.637
Race				
White	[reference]	--	[reference]	--
Black	0.88 (0.81–0.95)	0.002	0.91 (0.86–0.97)	0.002
Hispanic	1.07 (0.86–1.32)	0.573	1.11 (0.95–1.30)	0.174
Other	1.15 (0.90–1.47)	0.276	0.92 (0.77–1.10)	0.359
Injury Severity Score	1.08 (1.07–1.08)	<0.001	1.04 (1.04–1.04)	<0.001
Comorbidities				
None	[reference]	--	[reference]	--
One	0.89 (0.81–0.99)	0.027	0.91 (0.84–0.98)	0.009
Two	1.06 (0.94–1.21)	0.327	0.94 (0.86–1.02)	0.146
Three or more	1.02 (0.68–1.54)	0.929	0.88 (0.65–1.18)	0.379
Complications	1.52 (1.47–1.57)	<0.001	1.17 (1.14–1.21)	<0.001
Mechanism				
Penetrating	[reference]	--	[reference]	--
Blunt	1.57 (1.40–1.77)	<0.001	1.93 (1.77–2.10)	<0.001
ICU admission				
None	[reference]	--	[reference]	--
One or more days	3.22 (3.03–3.4)	<0.001	1.66 (1.57–1.76)	<0.001
Hospital length of stay	1.05 (1.04–1.05)	<0.001	1.01 (1.01–1.02)	<0.001
Insurance Type				
Commercial	[reference]	--	[reference]	--
Medicare	0.78 (0.70–0.87)	<0.001	0.70 (0.65–0.76)	<0.001
Medicaid	1.19 (1.01–1.40)	0.035	0.97 (0.86–1.08)	0.550
No Insurance	0.80 (0.72–0.88)	<0.001	0.86 (0.81–0.93)	<0.001
Government	0.63 (0.53–0.74)	<0.001	0.75 (0.66–0.85)	<0.001

In the adjusted model (Table 3), the RRs for the base model (all patients, *n* = 3261) indicate that insurance type was a statistically significant determinant of the total number of images received for all insurance groups in reference to those with commercial indemnity plans. Compared with the reference group, the effect of insurance status on the expected number of images patients received during their entire hospital stay ranged from a decrease of 10 % among the uninsured (0.90, 0.85–0.96) to 19 % among those with government insurance plans. During the first 24-hours of care, insurance status was attributed to a 22 % decrease in imaging among Medicare recipients (0.78, 0.71–0.86) and a 17 % decrease in imaging among those with government insurance plans (0.83, 0.74–0.94). Differences in specific CT imaging studies during the first 24-hours were observed among those having Medicare (0.74, 0.66–0.82) and government insurance plans (0.85, 0.74–0.97).

**Table 3:** Adjusted Risk Ratios (95 % CI) for type and frequency of diagnostic imaging for all adult patients and specific patient sub-groups

Insurance Type	All imaging	*p* value	Imaging within first 24 hours	*p* value	CT imaging within first 24 hours	*p* value
Base Model (3621)^a^						
Commercial	[reference]		[reference]		[reference]	
Medicare	0.85 (0.78–0.93)	<0.001	0.78 (0.71–0.86)	<0.001	0.74 (0.66–0.82)	<0.001
Medicaid	0.89 (0.81–0.99)	0.003	0.95 (0.85–1.06)	0.363	0.99 (0.87–1.12)	0.828
No Insurance	0.90 (0.85–0.96)	<0.001	0.96 (0.89–1.03)	0.239	1.01 (0.93–1.09)	0.788
Government	0.81 (0.72–0.90)	<0.001	0.83 (0.74–0.94)	0.003	0.85 (0.74–0.97)	0.019
Injury Severity Score > 15 (*n* = 596)^b^						
Commercial	[reference]		--		[reference]	
Medicare	0.86 (0.71–1.04)	0.122	0.85 (0.68–1.06)	0.145	0.84 (0.69–1.03)	0.093
Medicaid	0.78 (0.68–0.94)	<0.001	0.82 (0.66–1.03)	0.091	0.98 (0.80–1.20)	0.849
No Insurance	0.80 (0.71–0.91)	<0.001	0.84 (0.72–0.97)	0.016	1.02 (0.89–1.16)	0.785
Government	0.64 (0.51–0.81)	<0.001	0.72 (0.55–0.94)	0.016	0.88 (0.69–1.12)	0.283
Injury Severity Score < 15 (*n* = 3025)^b^						
Commercial	[reference]		--		[reference]	
Medicare	0.82 (0.74–0.91)	0.001	0.75 (0.67–0.84)	<0.001	0.71 (0.62–0.81)	<0.001
Medicaid	0.93 (0.82–1.05)	0.237	0.97 (0.84–1.11)	0.641	0.96 (0.82–1.13)	0.642
No Insurance	0.94 (0.87–1.01)	0.094	0.99 (0.91–1.07)	0.795	1.00 (0.91–1.10)	0.957
Government	0.84 (0.74–0.95)	0.007	0.85 (0.74–0.97)	0.021	0.83 (0.71–0.98)	0.027
Blunt force mechanism (*n* = 3058)^c^						
Commercial	[reference]		[reference]		[reference]	
Medicare	0.82 (0.76–0.89)	<0.001	0.77 (0.70–0.84)	<0.001	0.72 (0.65–0.79)	<0.001
Medicaid	0.82 (0.74–0.90)	<0.001	0.91 (0.82–1.02)	0.109	0.99 (0.88–1.12)	0.900
No Insurance	0.88 (0.83–0.94)	<0.001	0.94 (0.88–1.01)	0.085	1.01 (0.94–1.09)	0.795
Government	0.82 (0.74–0.91)	<0.001	0.85 (0.76–0.96)	0.006	0.85 (0.75–0.97)	0.015

^a^- adjusted for age, sex, race/ethnicity, injury severity score, injury mechanism, comorbidity, complications, and hospital LOS

^b^- adjusted for age, sex, race/ethnicity, injury mechanism, comorbidity, complications, hospital LOS, and ICU admission

^c^- adjusted for age, sex, race/ethnicity, injury severity score, comorbidity, complications, ICU admission, and hospital LOS

In the subgroup analysis among patients with an ISS > 15 (*n* = 596), all insurance plans other than Medicare were associated with a decrease in the expected number of images received during the entire hospital stay. This effect ranged from a 36 % decrease among the government insurance group (0.64, 0.51–0.81) to a 20 % decrease among the uninsured (0.80, 0.71–0.91). Differences in the total number of images received during the first 24-hours of care were observed only among the uninsured (0.84, 0.72–0.97) and government insurance groups (0.72, 0.55–0.94). No statistically significant differences were observed across insurance types in reference to five specific CT imaging types.

In the subgroup analysis among patients with an ISS < 15 (*n* = 3025), Medicare and government insurance plans were associated with a decrease in the expected number of images received during the entire hospital stay, the first 24 hours of care, as well as specific CT imaging received during the first 24 hours. Among Medicare recipients, this effect ranged from a 29 % decrease for specific CT scans during the first 24 hours (0.71, 0.62–0.81) to an 18 % decrease over the entire hospital stay (0.82, 0.74–0.91). Among government insurance recipients, this effect ranged from a 17 % decrease for specific CT scans during the first 24 hours (0.83, 0.71–0.98) to a 15 % decrease for total number of images within first 24 hours (0.85, 0.74–0.97). No statistically significant differences were observed across the uninsured or Medicaid recipients with ISS < 155.

In the subgroup analysis of blunt-injured patients (*n* = 3058), all insurance plans were associated with a decrease in the expected number of images received during the entire hospital stay. Compared to the reference group, the expected number of diagnostic images received during care decreased by 12 % among the uninsured and by 18 % among those with Medicare, Medicaid, or government insurance plans. Differences in the total number of images received during the first 24-hours of care were observed among Medicare (0.77, 0.70–0.84) and government insurance groups (0.85, 0.76–0.96). These differences were also found when assessed against specific CT imaging types.

Table 4 shows the adjusted RRs of the effect of insurance type on receipt of specific CT imaging procedures. Medicare insurance plans reduced the expected number of abdomen and pelvis with contrast imaging by 40 % (0.60, 0.48–0.65). Medicare and government insurance type decreased the number of chest/abdomen/pelvis with contrast screens by 45 % (0.55, 0.47–0.63) and 21 % (0.79, 0.66–0.94). No other insurance types were statistically significantly associated with an increase or decrease in the expected number of specific CT imaging types. Table 5 shows that there was no disparity in care among trauma patients who died in hospital overall or by more homogenous classifications of injury type or severity.

**Table 4:** Adjusted Risk Ratios (95 % CI) for type and frequency of selected computed tomography imagery by patient insurance type

	Computed Tomography type									
Insurance Type	Abdomen and pelvis w/contrast	*p* value	Chest/abdomen/pelvis w/contrast	*p* value	Cervical spine w/reconstruction	*p* value	Facial bones w/out contrast	*p* value	Head w/out contrast	*p* value
Base Model (*n* = 3621)^a^										
Commercial	[reference]		[reference]		[reference]		[reference]		[reference]	
Medicare	0.60 (0.48–0.65)	<0.001	0.55 (0.47–0.63)	<0.001	0.78 (0.67–0.90)	0.001	0.85 (0.64–1.12)	0.250	0.95 (0.85–1.07)	0.392
Medicaid	0.93 (0.79–1.09)	0.366	0.93 (0.79–1.09)	0.352	1.03 (0.87–1.22)	0.745	1.24 (0.92–1.67)	0.151	1.02 (0.89–1.17)	0.794
No Insurance	0.96 (0.87–1.05)	0.357	0.95 (0.86–1.05)	0.288	1.04 (0.94–1.15)	0.479	1.19 (0.99–1.43)	0.058	1.06 (0.98–1.16)	0.152
Government	0.78 (0.65–0.92)	0.004	0.79 (0.66–0.94)	0.008	0.92 (0.77–1.10)	0.339	0.87 (0.62–1.22)	0.407	0.92 (0.79–1.07)	0.261

^a^- adjusted for age, sex, race/ethnicity, injury severity score, injury mechanism, comorbidity, complications, ICU admission, and hospital LOS

**Table 5:** Adjusted Risk Ratios (95 % CI) for type and frequency of diagnostic imaging for patients who died while in hospital

Insurance Type	All imaging	*p* value
Base Model (128)^a^		
Commercial	[reference]	
Medicare	1.01 (0.57–1.79)	0.971
Medicaid	1.93 (0.92–4.03)	0.081
No Insurance	1.23 (0.73–2.06)	0.436
Government	1.42 (0.42–4.82)	0.570
Injury Severity Score > 15 (*n* = 97)^b^		
Commercial	[reference]	
Medicare	0.73 (0.30–1.77)	0.489
Medicaid	2.06 (0.85–4.99)	0.109
No Insurance	1.29 (0.71–2.37)	0.405
Government	0.95 (0.19–4.79)	0.949
Blunt force mechanism (*n* = 93)^c^		
Commercial	[reference]	
Medicare	1.05 (0.59–1.86)	0.874
Medicaid	1.84 (0.82–4.11)	0.137
No Insurance	0.86 (0.46–1.61)	0.637
Government	0.74 (0.13–4.28)	0.734

^a^- adjusted for age, sex, race/ethnicity, injury severity score, injury mechanism, comorbidity, complications, and hospital LOS

^b^- adjusted for age, sex, race/ethnicity, injury mechanism, comorbidity, complications, and hospital LOS

^c^- adjusted for age, sex, race/ethnicity, injury severity score, comorbidity, complications, ICU admission, and hospital LOS

## Discussion

Radiographic imaging is a routine component of trauma care and an important determinant of survival following trauma [32]. This study suggests that insurance status is a determining factor in delivery of imaging during trauma care, even after adjustment for clinical and demographic confounders. To our knowledge this is the first study to examine variation in radiographic imaging across multiple insurance types and across multiple time points among an entire trauma cohort while also controlling for demographic and clinical characteristics, including insurance status, injury severity, complications, comorbidities, injury mechanism and hospital stay. These results support ongoing investigations that insurance coverage is a determinant of variations in outcomes of trauma care.

Two key findings from this study stand out. First, our findings suggest that differences in care may be an artifact of the time period chosen for analysis. For example, we found fewer discrepancies during the acute phase of care, particularly in reference to specific CT imaging types. Similarly, no differences in care were observed among the uninsured and the Medicaid insurance group during the first 24-hours of trauma care, two populations widely recognized as receiving fewer operative procedures and experiencing poorer trauma outcomes. Second, our findings suggest that differences in care were not specific to the uninsured, but for all insurance groups in reference to patients with commercial indemnity plans. These findings are particularly alarming considering that equivalent care is mandated by law [33]. In particular, differences in the type and frequency of care received overall and during the acute phase of trauma care was consistently found among Medicare recipients. These findings remained after a sensitivity analysis which restricted the Medicare population to those aged 65 years or older (persons with disabilities are enrolled in Medicare after a waiting period, results not shown). However, outcomes among this patient group should be interpreted conservatively as we were unable to determine whether these differences could be explained by Medicare plan type (e.g. Part A, Part B).

Insurance status may have affected the type and frequency of procedures patients in our hospital received for several reasons. First, discretionary care may have been provided based on an individual’s ability to pay. For example, there were distinct similarities between results from this study and those of the Bolorunduro et al. [29] and Haas and Goldman’s [27] study comparing type and frequency of procedures delivered during trauma care, as all findings suggest that lack of insurance was a significant determining factor in receipt of diagnostic imaging after controlling for other demographic and clinical factors. Although direct comparisons are difficult due to subtle differences in methodology and patient mix between studies, considering that all studies demonstrated a significant association between lack of insurance and the number of therapeutic procedures received reflects the underlying relationship between socioeconomic status and health outcomes. However, out study findings also show that these differences may be reduced and in some instances eliminated if the analysis is partitioned into different time periods.

Second, differences in care could have occurred due to physician bias. Although physicians may not perceive disparities in their own care, previous studies have shown that racial stereotypes play a role in determining the type and frequency of therapeutic options physicians prescribe for treatment [34, 35]. However, in the adjusted model race/ethnicity did not remain a statistically significant determinant of differences in radiographic imaging. In part, our subgroup analysis was designed to determine whether variations in care could be attributed to a specific time period or injury type, thus identifying areas for further audit. For example, one would expect that the multi-system trauma patient would require more imaging not only in the first 24 hours, but throughout their ICU stay, whereas those with minor injuries would require less imaging and testing. However, the subgroup analyses confirmed the overall trend in variations in care.

A third reason could be attributed to patient health literacy [36]. In particular, uninsured patients are known to experience difficulties accessing and communicating with physicians [37]. However, this explanation may be difficult to isolate here given that differences in care were identified across all insurance groups. Lastly, it is also possible that variations in care were attributed to resource limitations. For example, over 30 % of the patient population in this study was uninsured, which was more than twice the national rate during this period [38].. Again, this explanation may be difficult to isolate given that differences were observed across all insurance types and were often less pronounced among the uninsured patient population. Although we can only speculate as to its effect, our results may suggest at some point during a patient’s stay there is either discretionary care based on physician bias, a patient’s ability to pay, or that patients are electing to decline procedures that may not be reimbursable.

These results should be interpreted with respect to three key limitations. First, due to limitations of coding in the trauma registry, we were unable to identify patients who had more than three comorbidities. As a result, this may have inflated the association between Medicare insurance status and the number of images received during care. Second, we were unable to control for the type of Medicare plan (e.g. Part A, Part B) patients were enrolled in, which may also help to explain the observed differences in care, particularly if patient’s were electing not to receive a procedure due to co-payment costs. Third, while we excluded patients who were discharged to prison from our analysis, we were unable to verify if the entire Government insurance cohort was specific to those who had military or Tricare insurance plans.

Current changes to US health care policy through the ACA as well as state determination for legislating Medicaid expansion to lower-income groups mandate more rigorous and frequent investigation of disparities in outcomes from trauma care owing to insurance status. At the time of this study, South Carolina was one of 16 states that elected not to lower the eligibility requirements for enrollment [39]. Based on findings from this study, improvements in health care coverage in the state will unlikely eliminate social inequalities among the injured. Given our findings that the Medicaid and the uninsured populations receive fewer interventions overall while in care suggests that improvement in the monitoring and evaluation of care practices is needed. Understanding the mechanisms that lead to differences in care is an area for future work.

## Conclusion

To date, most research on disparities in the delivery of diagnostic imaging studies among the major trauma patient has been specific to outcomes among the uninsured. However, we found notable differences in care across numerous insurance groups as well as the uninsured in comparison to those with commercial indemnity plans. These findings support routine evaluation of care practices among all patient groups to improve the quality of trauma care. Our findings also illustrate that analyzing the effect of insurance status on the type and frequency of diagnostic images that patients receive, without taking into account the specific time when interventions were delivered, influences the interpretation of inequities in trauma care. In particular, there were no statistically significant differences in the type or frequency of radiographic imaging received by the uninsured or Medicaid patients during the first 24-hours of trauma care, but notable differences in treatment were observed among patients with ISS > 15.

## Abbreviations

ACA, Affordable Care Act; ACS, American College of Surgeons; ISS, injury severity score; LOS, length of stay; MIAP, medically indigent assistance program; PHRH, Palmetto Health Richland Hospital
